# Successful management of trichotillomania in a patient with SSRI-induced hypersomnia: a case report

**DOI:** 10.3389/fpsyt.2026.1938337

**Published:** 2026-08-20

**Authors:** Deemah Ateeq Alateeq

**Affiliations:** Internal Medicine Department, College of Medicine, Princess Nourah bint Abdulrahman University, Riyadh, Saudi Arabia

**Keywords:** bupropion, case report, hypersomnia, SSRI intolerance, trichotillomania

## Abstract

**Background:**

Trichotillomania is predominantly managed via serotonergic pharmacotherapy and behavioral interventions. However, options become scarce when patients exhibit extreme intolerance to first-line agents. Bupropion, a norepinephrine-dopamine reuptake inhibitor (NDRI), has been reported in a limited number of cases as a potential treatment option for hair-pulling disorders but remains infrequently used in clinical practice.

**Case presentation:**

A 25-year-old female medical student with a 13-year history of severe, stress-triggered Trichotillomania developed debilitating hypersomnia (16–18 hours of sleep/day) during separate trials of fluoxetine and escitalopram. This adverse effect, also historically reported by her mother, forced abrupt drug cessation and caused severe academic decline during medical school. Following intolerance to two selective serotonin reuptake inhibitors (SSRIs), the patient was treated with a combination of bupropion and therapist-guided mindfulness-based behavioral therapy. Over the subsequent two months, she experienced marked improvement in hair-pulling behaviors without recurrence of hypersomnia. Clinical improvement was sustained throughout a 24-month follow-up period after discontinuation of bupropion while continuing intermittent self-directed mindfulness practice.

**Conclusion:**

This case suggests that a combined treatment approach incorporating bupropion and mindfulness-based behavioral therapy may be associated with sustained improvement in trichotillomania when SSRIs are poorly tolerated. Although the individual contribution of each intervention cannot be determined from a single case, this report adds to the limited literature supporting further investigation of non-serotonergic pharmacological strategies in selected patients.

## Introduction

Trichotillomania (hair-pulling disorder) is a chronic body-focused repetitive behavior characterized by the recurrent pulling out of one’s hair, resulting in significant functional impairment, emotional distress, and visible alopecia. The neurobiology of Trichotillomania is complex, but therapeutic strategies have traditionally focused on modulating the serotonergic system. Consequently, selective serotonin reuptake inhibitors (SSRIs) are frequently prescribed as first-line pharmacotherapy, despite mixed and conflicting evidence regarding their long-term efficacy when utilized in isolation without specialized behavioral modalities (1).

While SSRIs are generally well-tolerated, they can significantly disrupt baseline sleep architecture. Although insomnia is widely recognized as a classic side effect, treatment-limiting hypersomnia or profound, daytime somnolence remains a rarer and poorly understood adverse event that severely threatens patient compliance and treatment persistence (2). When severe intolerance or atypical idiosyncratic reactions preclude the use of standard serotonergic agents, clinicians lack established algorithmic guidelines to steer alternative pharmaceutical routes.

Although serotonergic therapies have historically received the greatest attention, emerging evidence suggests that dysregulation of dopaminergic and noradrenergic pathways within cortico-striato-thalamo-cortical circuits may also contribute to the pathophysiology of trichotillomania. Consequently, medications that modulate these neurotransmitter systems have been explored as potential alternatives in selected patients, although the supporting evidence remains limited (3–5).

Bupropion, a norepinephrine-dopamine reuptake inhibitor (NDRI), is primarily indicated for major depressive disorder and smoking cessation, but is traditionally overlooked or omitted in standard obsessive-compulsive spectrum treatment profiles due to its lack of direct serotonergic activity. However, growing evidence points to alternate neural circuit networks in impulse mitigation. This case report describes the successful management of severe, chronic trichotillomania using a combined treatment approach consisting of bupropion and mindfulness-based behavioral therapy in a patient with severe SSRI-induced hypersomnia. The case adds to the limited literature describing the use of bupropion in trichotillomania, particularly in the context of marked SSRI intolerance (3–5).

## Case presentation

### Patient history

A 25-year-old female medical student presented with a 13-year history of trichotillomania targeting frontal and vertex scalp regions since age 12, resulting in 5x5 cm alopecia patches, causing profound academic and social isolation. Symptoms escalated dramatically during medical school, becoming daily, focused episodes lasting over 30 minutes, heavily triggered by exam stress. The diagnosis of trichotillomania was established by a consultant psychiatrist based on the Diagnostic and Statistical Manual of Mental Disorders, Fifth Edition (DSM-5) criteria. Clinical assessment did not identify any current or past psychiatric comorbidities, including depressive, anxiety, obsessive-compulsive, psychotic, or substance use disorders. The patient had not previously received structured psychotherapy or behavioral interventions for trichotillomania prior to the current treatment episode. Standardized symptom severity scales were not administered because treatment occurred during routine clinical practice rather than within a research protocol. Clinical improvement was based on serial psychiatric assessments and patient reports.

### The failures (SSRI intolerance trials)

Trial 1 (22-year-old): Fluoxetine initiated at 20 mg/day and titrated to 40 mg/day over 6 months. Induced profound hypersomnia (16–18 hours/day), forcing abrupt self-discontinuation due to academic failure.

Trial 2 (23-year-old): Escitalopram 10 mg/day trial over 5 months. Replicated identical severe somnolence, leading to another self-discontinuation. Patient noted her mother experienced an identical hypersomnic profile on fluoxetine.

### The third therapeutic intervention (25-year-old)

A third treatment strategy was implemented following a formal psychiatric consultation. Given the patient’s documented history of severe intolerance to multiple serotonergic agents, the clinical team elected to completely bypass the SSRI pathway. Pharmacotherapy was initiated with bupropion at 150 mg once daily for 1 week, which was subsequently titrated to a maintenance dose of 300 mg once daily. Although the patient initially preferred exclusive psychological intervention due to her prior negative experiences with medications, she consented to this alternative pharmacotherapeutic strategy after a detailed clinical discussion regarding its distinct, non-serotonergic mechanism of action. Concurrently, the patient engaged in a structured psychological regimen consisting of therapist-guided, mindfulness-based behavioral interventions. This therapeutic course consisted of five therapist-guided mindfulness-based behavioral sessions delivered over approximately 10 weeks at two-week intervals, followed by continued independent practice by the patient.

### Clinical course

Within two months of initiating the combined bupropion and mindfulness-based protocol, the patient reported progressive improvement in her ability to control hair-pulling behaviors and to successfully suppress the compulsive urge to pull. This was accompanied by a significant reduction in the frequency of pulling episodes, culminating in successful management by the end of the second month.

Crucially, this period of clinical remission coincided with, and persisted through, a phase of severe, compounding psychosocial stressors that manifested after the initiation of treatment. These subsequent stressors included her medical school graduation, the transition to intensive internship training, preparation for the high-stakes Medical Licensing Examination, and active participation in competitive residency matching interviews. Despite the psychological toll of these consecutive milestones, no recurrence of hair-pulling behavior was observed, demonstrating robust therapeutic resilience.

Pharmacological therapy was maintained for approximately 7 months. It was eventually discontinued abruptly by the patient due to sudden medication unavailability. Throughout the treatment course, the only adverse effect reported was bilateral hand tremor. This tremor specifically affected her fine motor control during surgical practice, raising clinical notice given her medical training. Notably, this side effect fully resolved following the discontinuation of bupropion.

### Follow-up and outcomes

Crucially, from the initial onset of the disorder at age 12 until the implementation of bupropion at the age of 25, the patient had never experienced a hair-pulling-free interval lasting longer than two months, underscoring the severe and chronic nature of her condition.

At the time of writing, the patient has maintained successful management for 24 months, remaining entirely free of hair-pulling behavior. Notably, this prolonged clinical remission has been achieved and maintained without ongoing pharmacological therapy following bupropion discontinuation.

Furthermore, the patient reports continuous, robust regrowth of scalp hair, with complete resolution and absence of recurrent alopecia patches. Throughout this 24-month follow-up period, she has independently maintained intermittent, self-directed mindfulness practices, which have served as her primary non-pharmacological strategy for long-term relapse prevention. A summary of the patient’s clinical timeline is presented in **Table 1**.

**Table 1:** Clinical timeline of the patient’s diagnosis, treatment, and follow-up.

Age	Clinical event	Intervention	Duration	Outcome
12 years	Onset of trichotillomania affecting frontal and vertex scalp	None	13 years	Persistent hair-pulling with recurrent alopecia
22 years	First psychiatric treatment	Fluoxetine 20–40 mg/day	6 months	Severe hypersomnia (16–18 h/day); treatment discontinued
23 years	Second pharmacological trial	Escitalopram 10 mg/day	5 months	Recurrent severe hypersomnia; treatment discontinued
25 years	Psychiatric reassessment (DSM-5 diagnosis confirmed)	Bupropion 150 mg/day for 1 week, increased to 300 mg/day, plus therapist-guided mindfulness-based behavioral therapy (five sessions every 2 weeks)	Bupropion: ~7 months; Mindfulness therapy: 10 weeks	Progressive reduction in hair-pulling within 2 months; sustained remission; no hypersomnia; mild hand tremor during treatment
25 years	Medication discontinued because of unavailability	Continued intermittent self-directed mindfulness practice	Ongoing	Tremor resolved after stopping bupropion
27 years	Follow-up	No pharmacological treatment	24 months after remission	Sustained remission with complete hair regrowth and no recurrence

## Discussion

The successful management of this patient’s trichotillomania following combined treatment with bupropion and mindfulness-based behavioral therapy provides additional clinical experience supporting alternative therapeutic approaches for patients who cannot tolerate SSRIs. Because both interventions were initiated concurrently, it is not possible to determine the relative contribution of bupropion and psychological therapy to the observed improvement. Therefore, the favorable outcome should be interpreted as the result of a combined treatment approach rather than as evidence of bupropion’s efficacy alone.

Although SSRIs remain among the pharmacological options used for trichotillomania, increasing evidence suggests that dysregulation of cortico-striato-thalamo-cortical (CSTC) circuits and dopaminergic pathways may also contribute to the disorder. Because bupropion enhances dopaminergic and noradrenergic neurotransmission, it has been hypothesized that it may influence neural circuits involved in impulse control and habit formation. However, the present case cannot establish this mechanism, and the observed clinical improvement should not be interpreted as evidence of causality (3).

Because pharmacotherapy and mindfulness-based behavioral therapy were initiated concurrently, the independent contribution of each intervention cannot be determined. Behavioral therapy is an established component of trichotillomania management and likely contributed to the favorable outcome. Conversely, the absence of hypersomnia during bupropion treatment may have improved treatment adherence and facilitated engagement with psychotherapy. Although expectancy or nocebo effects cannot be entirely excluded, particularly given the patient’s awareness of her mother’s adverse reaction to fluoxetine, the recurrence of profound hypersomnia during two separate SSRI trials and the sustained long-term remission observed in this case suggest that the clinical course is unlikely to be explained solely by such effects.

Furthermore, this case highlights severe hypersomnia as an uncommon but clinically important adverse effect of SSRI treatment that substantially affected treatment adherence and academic functioning. The occurrence of a similar reaction in the patient’s mother may suggest an underlying familial susceptibility to SSRI-induced hypersomnia. Potential pharmacogenetic mechanisms, including variation in CYP2D6 or CYP2C19 metabolism, have been proposed in the literature; however, no pharmacogenetic testing was performed in this patient, and such explanations remain speculative (6). The absence of hypersomnia during bupropion treatment is consistent with its pharmacological profile but should not be interpreted as evidence of superiority over serotonergic agents.

## Conclusion

This case adds to the limited literature describing the use of bupropion in patients with trichotillomania who are unable to tolerate SSRIs. The sustained clinical improvement observed following combined treatment with bupropion and mindfulness-based behavioral therapy suggests that this approach may be considered in carefully selected patients when first-line serotonergic therapy is not feasible. However, because both interventions were initiated concurrently, the contribution of each treatment cannot be determined. Further studies are needed to better define the role of bupropion within multimodal treatment strategies for trichotillomania.

## Patient perspective

Living with trichotillomania for more than 12 years had a significant impact on my academic performance, confidence, and social life. Previous treatment with SSRIs was difficult because of severe excessive sleepiness, which made me reluctant to try medication again. My psychiatrist listened to my concerns and involved me in choosing an alternative treatment with bupropion alongside mindfulness-based therapy. Within two months, I gradually regained control over my hair-pulling urges, and I have now remained free from hair-pulling for 24 months. This treatment has greatly improved my quality of life and has allowed me to successfully complete major milestones in my education and medical career without relapse.

## Data Availability

The original contributions presented in the study are included in the article/supplementary material. Further inquiries can be directed to the corresponding author.
